# Inferring the connectivity of coupled chaotic oscillators using Kalman filtering

**DOI:** 10.1038/s41598-021-01444-7

**Published:** 2021-11-17

**Authors:** E. Forero-Ortiz, G. Tirabassi, C. Masoller, A. J. Pons

**Affiliations:** Departament de Física, Universitat Politècnica de Catalunya, St. Nebridi 22, 08222 Terrassa, Barcelona Spain

**Keywords:** Complex networks, Nonlinear phenomena

## Abstract

Inferring the interactions between coupled oscillators is a significant open problem in complexity science, with multiple interdisciplinary applications. While the Kalman filter (KF) technique is a well-known tool, widely used for data assimilation and parameter estimation, to the best of our knowledge, it has not yet been used for inferring the connectivity of coupled chaotic oscillators. Here we demonstrate that KF allows reconstructing the interaction topology and the coupling strength of a network of mutually coupled Rössler-like chaotic oscillators. We show that the connectivity can be inferred by considering only the observed dynamics of a single variable of the three that define the phase space of each oscillator. We also show that both the coupling strength and the network architecture can be inferred even when the oscillators are close to synchronization. Simulation results are provided to show the effectiveness and applicability of the proposed method.

## Introduction

It is well-known that many real-world systems can be modelled as networks of interacting subsystems^1^. Common examples span from social sciences^2^ to biology^3^, ecology^4,5^, and climatology^6,7^, among others. Even through the types of networks and dynamical behaviours that exist in nature are highly diverse, difficult to observe experimentally, to model and to characterize^8–13^, network analysis has demonstrated to be an extremely useful technique for advancing our understanding of many complex systems. It is by now well known that identifying the connections between the various components of networks (isolated, multi-layer, networks of networks, etc.) may provide valuable insights towards understanding the structure and function of these systems. However, detecting interactions between the elements from the analysis of their observed dynamics is a challenging task, as genuine interactions can be masked by the presence of noise, indirect connections, common forcing or the existence of unobserved variables^14,15^.

In the past years, several techniques have been proposed to infer interactions and reconstruct the network topology by analyzing the evolution of the individual subsystems represented by the network nodes. These techniques can either be invasive—that is, require perturbing the system dynamics^16–18^—or non-invasive—that is, are based on the sole observation of the dynamics—. Many studies have been carried out applying cross-correlation and/or mutual information^19–23^, mutual information rate^24^, Granger causality^25^, partial directed coherence^26^, just to name a few.

Other techniques require some previous knowledge about the system’s elements^27–29^. In this work, we will follow this second paradigm. In particular, we will show that in the case of a system composed of coupled Rössler-like chaotic oscillators, one can retrieve the coupling constant and the adjacency matrix of the network with a high degree of accuracy by employing the Kalman Filter (KF) technique^30,31^.

The KF technique is commonly used as a data assimilation method^32^ where predictions coming from a known model of the system (the so-called prediction step) and experimental observations of the modelled system are integrated (the correction step) to obtain reliable estimates of the system state. However, by assuming that model parameters are also variables with trivial dynamics (they are constant), KF can also be used to retrieve their values [see^33^ and references therein]. Common applications of the Kalman filter involve smoothing noisy data and providing estimates of relevant parameters for diverse purposes such as weather prediction^34^, target tracking^35^, global positioning system receivers^36^, speech dynamics^37^, ocean chlorophyll estimation from satellite observations^38^, to name just a few. Whereas the Kalman filter has been widely used in the literature to find model parameters that optimally fit the observed data, to the best of our knowledge it has not yet been used for dynamic network and synchronisation analysis.

From this perspective, inferring the connectivity between two or more oscillators can be thought of as recovering the value of the parameters representing the coupling strengths: if the coupling strength between two nodes is different from zero, the oscillators are connected; otherwise, they are not. Here, as a first step, we show that KF can indeed infer the coupling strengths in a network of Rössler-like chaotic oscillators. To do this, we numerically simulate Rössler-like chaotic oscillators as described in Sevilla-Escoboza et al.^39^, and use the so-called *Unscented Kalman Filter* (UKF)^40^ implemented in *Matlab*.

Our motivation to use the expression of the Rössler-like chaotic oscillators given in^39^ is the existence of a large and freely available experimental data set (available for Ref.^39^and for Ref.^41^). However, as we will discuss later, the temporal filtering performed by the experimental data acquisition system prevents obtaining a good network reconstruction. Therefore, here, as a proof-of-concept demonstration, we limit our study to consider synthetically-generated time series. Future work will attempt to incorporate the data acquisition process into the method presented here, to try to reconstruct the network topology using the available experimental data.

In this study, we know the details of the governing equations of each Rössler-like oscillator and leave their coupling strengths as the only unknown parameters. We first test the algorithm with an isolated oscillator and show that, from the analysis of one of the variables, it is possible to retrieve the evolution of the other two variables which describe the system. Next, we consider two mutually coupled oscillators. For these two elements, first, we assume that the coupling is symmetric and we demonstrate that we can recover the strength of their coupling, *K*. In a second scenario, we do not assume symmetric coupling, and we recover the values of two coupling parameters, $K_{12}$ and $K_{21}$ (which should be identical). Then, we show that for 28 randomly coupled Rössler-like oscillators, the UKF technique can reconstruct the network architecture, inferring with high precision the presence or absence of links between pairs of oscillators. We investigate the quality of the network reconstruction across a wide range of values of coupling strength and show that we can accurately infer the network topology and the strength of the couplings, even when the oscillators are close to synchronization.

This paper is organized as follows. The UKF algorithm and the model of the chaotic Rössler-like oscillators used in this study are described in section “Methods”; in section “Results” we perform different demonstrations using the algorithm to prove that we are able to characterize the topology of networks using noisy data. In section “Discussion” we present our conclusions and discuss the advantages and limitations of this technique.

## Results

### Uncoupled dynamics: estimation of the unobserved oscillators’ variables

**Figure 1:**
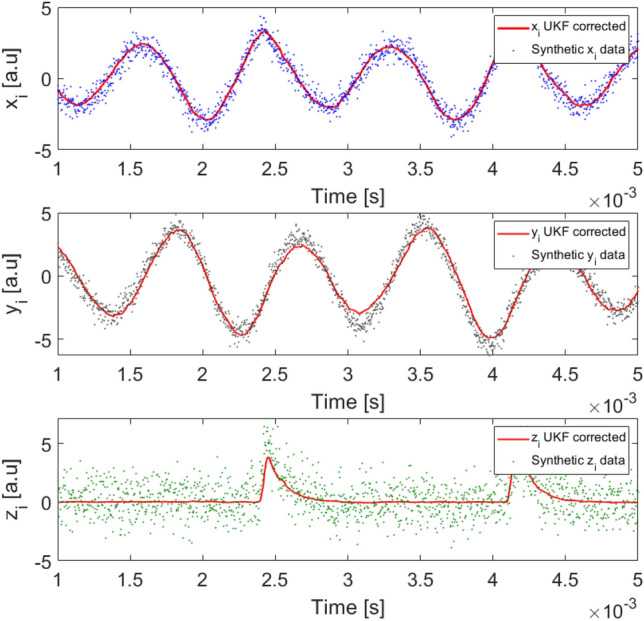
Raw simulated data and Kalman filter adjustment of the state variables $x_i(t)$, $y_i(t)$ and $z_i(t)$ of an isolated Rössler-like oscillator. The red lines are the estimation output from the UKF algorithm observing only the time series $y_i$. The parameters values used to generate the synthetic time series and the filtered adjustment are listed in Table 1. The points represent simulated data obtained by solving Eqs. (7a–c) and contaminated by the stochastic and noise terms (see Eqs. 3, 4). However, only the $y_i$ dataset is used as input to the UKF algorithm. The solid lines represent the values returned by the UKF algorithm.

We begin by demonstrating a simple application of the UKF technique. We consider a synthetic data set corresponding to an isolated, uncoupled oscillator. We set $N=1$ in Eqs. (7a–c) and solve the equations as described in “Methods” using the parameters listed in Table 1. Both, the numerically generated noisy values of the variables *x*, *y*, and *z* and the filtered time series obtained as described below are shown in Fig. 1. We remark that the UKF algorithm assumes that the input data are noisy measurements of an stochastic dynamical system, i.e., the input data is generated by a dynamically noisy system and it is contaminated by observational noise.

As expected, the UKF filters out the noise, recovering the smooth dynamics of the oscillator. This can be done even with high levels of observation noise (see Eq. 4). Therefore, the reconstruction of the evolution of the three variables defining the state of the system was performed using only the noisy time series of the *y* variable. The algorithm may also be applied to observations of the other variables, x or z (i.e., from noisy observations of x we can reconstruct the evolution of the unobserved y and z). This confirms that the algorithm can infer the full state of the system from partial information of its dynamics. This feature, which is at the heart of the KF technique, introduces high versatility to the method presented here for systems for which the observations are only partial and noisy.

### Minimal motif: two coupled oscillators

We now expand our analysis to a minimal motif formed by a system composed of two coupled oscillators. We set $N=2$ and simulate Eqs. (7a–c) using a value of the coupling strength *K* that we will try to recover, from the analysis of the time series of the variables $y_1$ and $y_2$ of the two oscillators.

**Figure 2:**
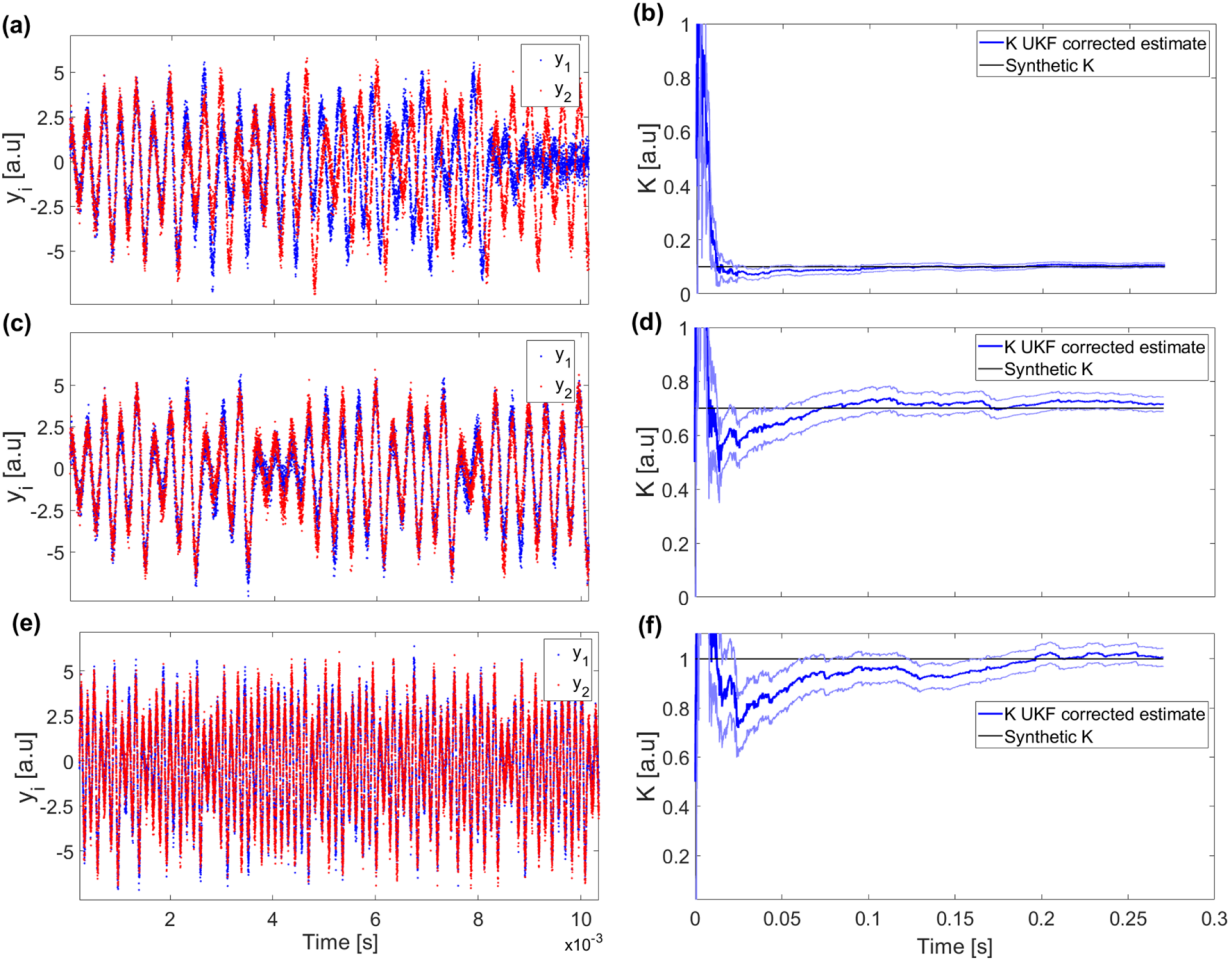
Left panels show the time series of $y_1(t)$ and $y_2(t)$ for two coupled nodes with strengths $K=0.1$ (**a**), $K=0.7$ (**c**) and $K=1.0$ (**e**). Notice that in panels (**a**) and (**c**) the evolution of the variables shows some interaction but not fully synchronization, which is illustrated in panel (**c**). Right panels, (**b**), (**d**) and (**f**), respectively, show the evolution of the UKF estimation of the parameter K for each group of synthetic data shown of its left. The solid black line indicates the value of the synthetic K to be obtained by the UKF estimation. The blue line is the estimation output from the UKF. The blue lighter lines indicate the $\sigma$ and $-\sigma$ uncertainty bands defined by the square root of the estimate of the state covariance matrix for the parameter value K. The parameters used for these simulations and filtering are given in Table 1. Their duration is 100,000 time steps.

To estimate *K*, we extend the UKF state as shown in Eq. (5). We generated synthetic $y_1$ and $y_2$ measurement data time series for different coupling strengths *K*. In Fig. 2a, for example, we plot the time evolution of variables $y_1$ and $y_2$ when $K=0.1$. It can be seen that the oscillators are significantly coupled, but not fully synchronized. From these two time-traces, we can estimate both, the state of the two oscillators and *K*.

In order to apply the UKF, we prescribe^33^ the model covariance $7\times 7$ matrix, ${\bar{Q}}_{\omega , e}$, (see Eq. 6) to be

1 $${\bar{Q}}_{\omega , e} = \begin{pmatrix} {\bar{Q}}^{\omega } &{} 0 \\ 0 &{} 0 \end{pmatrix}$$

where ${\bar{Q}}^{\omega }$ is a covariance matrix estimated using a realization of a Gaussian process with $\sigma _{\omega }=0.02$, and the $2\times 2$ measurement covariance ${\bar{Q}}_{\nu }$ is estimated using a realization of a Gaussian process with $\sigma _{\nu }=0.5$ (see Table 1). To run the UKF algorithm, a priori, we do know the exact value of the covariances. However, ${\bar{Q}}_{\omega }$ covariances created from $\sigma _{\omega }$ ranging from 0.001 to 5 and ${\bar{Q}}_{\nu }$ covariances created from $\sigma _{\nu }$ ranging from 0.001 to 1 have been tested. In all cases the algorithm converges to the correct value of *K*, showing its robustness to initial guesses.

**Figure 3:**
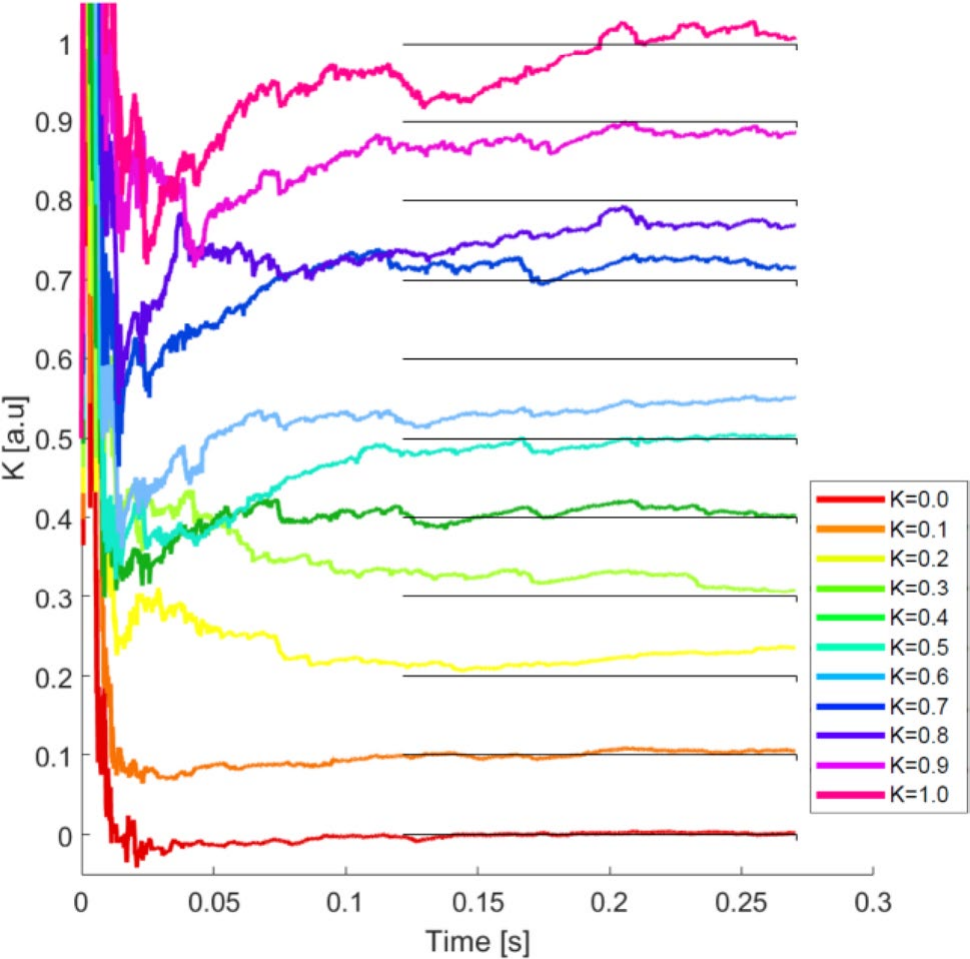
UKF estimations obtained from the observations $y_1$ and $y_2$ for *K* parameters ranging from 0 to 1. The solid black lines indicate the values of the K parameters to be estimated during the filtering. The estimated values converge, in all cases, independently of the levels of synchronisation.

In Fig. 2b, the evolution of the estimate of *K* is shown. In this panel, we see that the real coupling strength (0.1) is obtained after a few iterations of the algorithm. Figure 2c shows the time evolution of variables $y_1$ and $y_2$ when $K=0.7$. In this case, the evolution of both elements is partially synchronized. The inference of *K* is shown in Fig. 2d. The parameter is accurately estimated (blue line) with less than $0.1\%$ relative error, and the estimation is within one standard deviation (within the blue lighter lines) before 1/3 of the time series has been analysed. For the case of full synchronisation shown in Fig. 2e, we see that the algorithm, after a transient, also converges to the correct value (see Fig. 2f). The ability of the UKF algorithm to infer the correct value of the parameter despite the high level of synchronization of the two oscillators is a remarkable property worth noting.

This result confirms the ability of the UKF to recover correctly a single value of *K*. However, one may argue, as results shown in Fig. 2 seem to suggest, whether different levels of synchronization might make it more challenging to correctly estimate the coupling (specifically, from the noisy and partial measurement of the states of the oscillators). Therefore, to test the robustness of the UKF method, we created synthetic data sets for different values of *K* ranging from 0 to 1. The evolution of the estimations of the parameter *K* for these data sets is shown in Fig. 3. As it can be seen, the UKF is able to give a good estimate across the whole range of *K* values. We speculate that, if the length of the data-set used is increased, the result of the estimate will converge to the neighborhood a fixed value. One can also notice that the convergence takes more time as *K* increases. In all cases, the value of $K=0.5$ was selected as the initial estimate because this is the value which is in the middle of the parameter range $K=0-1$. Since the initial guess for *K* is always the same, the reason for the increasingly long transient is not an increasing distance to the real value of *K*, thus we wonder that the longer transient may be related to the synchronization level of the signals. The parameter convergence depends on this initial estimate, however, the impact in our system of the initial estimate is minimal and does not significantly affect the parameter estimation performance or output.

**Figure 4:**
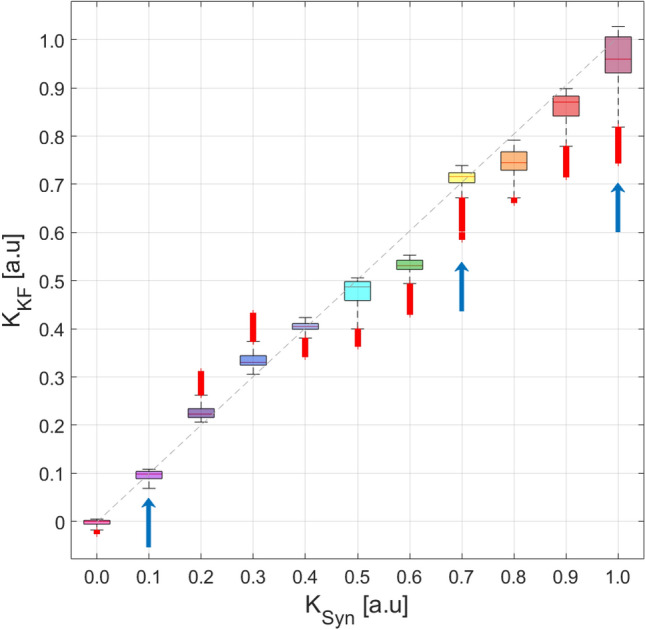
UKF estimation of the parameter *K* versus the real value. The comparison is made using boxplots. For each boxplot, the red line is the median, the upper and lower whiskers of the bars represent the maximum and minimum limits, and the red points refer to outliers. For the construction of each boxplot, we use 90.000 data points obtained from independent runs of the UKF algorithm for each synthetic data set. The dashed-black diagonal line indicates the value where the real K and the estimated K are the same. Blue arrows correspond to the results shown in Fig. 2.

Figure 4 shows boxplot distributions of the UKF estimated *K* values versus the true *K* values used to generate the synthetic time series for $y_1$ and $y_2$. To generate the figure, we considered the last 90,000 of the 100,000 data points obtained by the UK filter process. It is worth noting that the estimation for $K=0.6$ is significantly away from the optimal result, while the estimates for the other *K* values are reasonably close to the target values (see also Fig. 3). In general, we can observe a worse agreement in the case in which the coupling is relatively high, where the oscillators start synchronizing.

### Coupling directionality

So far, we assumed that the oscillators are symmetrically coupled (our prior knowledge is that the adjacency matrix is symmetric). We now relax this assumption by defining two coupling constants: $K_{12} = K\cdot A_{12}$ and $K_{21} = K\cdot A_{21}$. By doing so, we check that the method can detect the directionality of the coupling.

**Figure 5:**
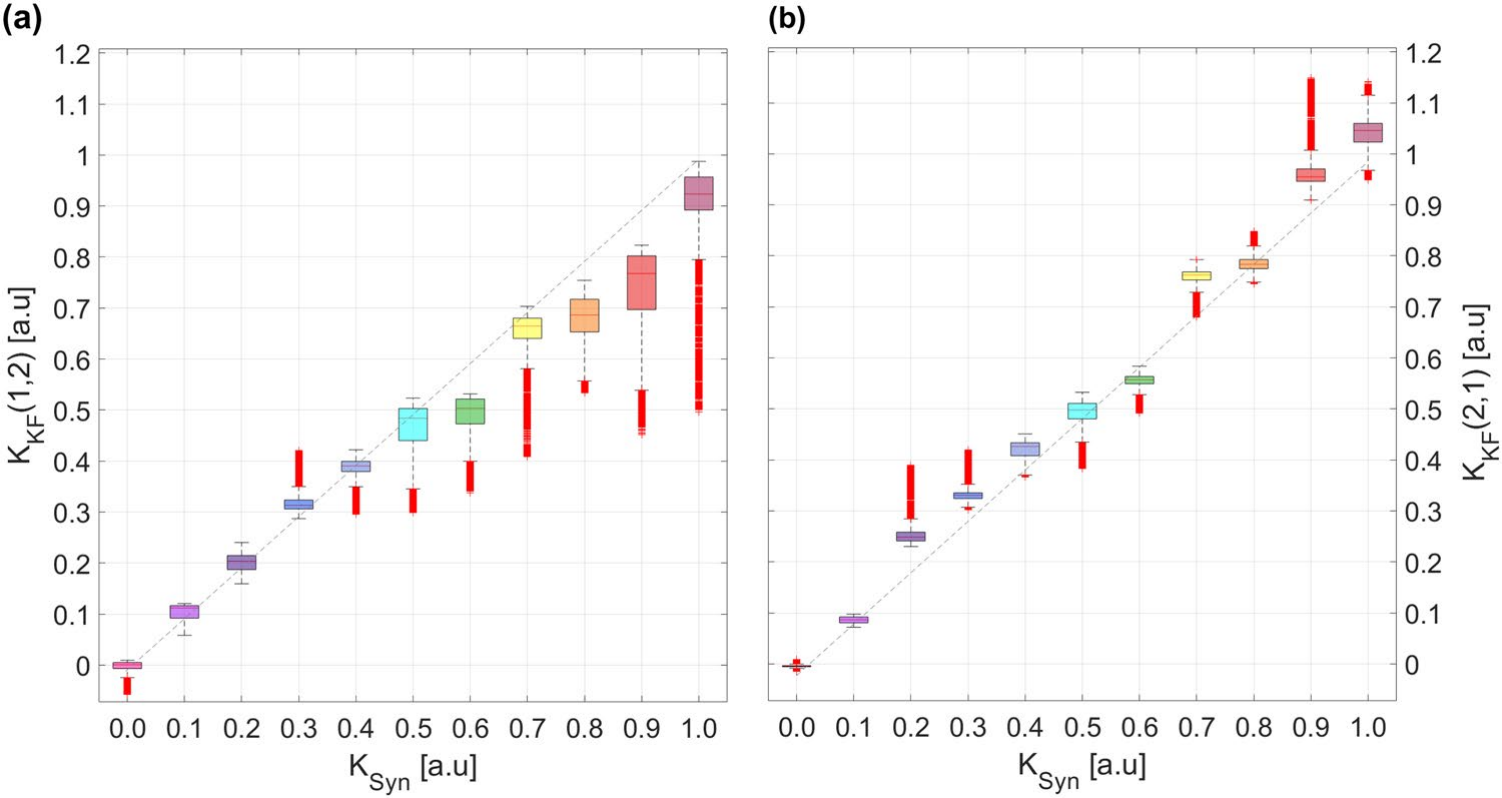
Comparison between the UKF estimated product of the off-diagonal adjacency matrix terms, $A_{12}$ and $A_{21}$, times the coupling strength, *K* ($K_{12} \equiv K\cdot A_{12}$ and $K_{21} \equiv K\cdot A_{21}$) versus the real value *K*. The comparison is made using boxplots. (**a**) Comparison for $K_{12}$. (**b**) Comparison for $K_{21}$. For each boxplot, the red line is the median, the upper and lower whiskers of the bars represent the maximum and minimum limits, and the red points refer to outliers. Each box contains 90,000 data obtained from independent runs of the UKF algorithm for each synthetic KA-parameters. The dashed-black diagonal line indicates the value where the synthetic KA and the estimated KA are the same.

We see in Fig. 5 that the UKF algorithm provides reliable estimates of $K_{12}$ and $K_{21}$, but the estimates are more scattered, in comparison of the estimation shown in Fig. 4. Even though the UKF algorithm tends to slightly underestimate (overestimate) the true $K_{12}$ ($K_{21}$) values, it provides good estimates for a wide range of *K*s. One may note that the whiskers cover the diagonal dashed line in most cases, meaning that the estimates of the parameters are statistically valid. While we don’t know why the estimates of the parameters $K_{12}$ and $K_{21}$ are more dispersed, we speculate that it may be due to the fact that we are estimating two parameters ($K_{12}$ and $K_{21}$) instead of one (*K*), which possibly requires longer datasets for the parameters to converge, and/or the algorithm may be more sensitive to small fluctuations. Further tests are needed to determine how the sensitivity of the algorithm depends on the number of estimated parameters, the amount of data, the computational power required, and the quality of the results obtained.

### Network of 28 randomly coupled Rössler-like chaotic oscillators

Lastly, we tackled the analysis of a network of $N=28$ oscillators. We simulate Eqs. (7a–c) using a known value of the coupling strength *K* and a known adjacency matrix $A_{ij}$ (Network 1 in ref.^41^). In this way, we obtain a synthetic data set with 28-time series ($y_i$ with $i=1\dots 28$) which we use to first try to recover *K* (assuming that the adjacency matrix $A_{ij}$ is known) and then, we try to recover all the 378 ($N(N-1)/2$) values of $K\cdot A_{ij}$, assuming that $A_{ii}=0$ and $A_{ij}=A_{ji}$.

**Figure 6:**
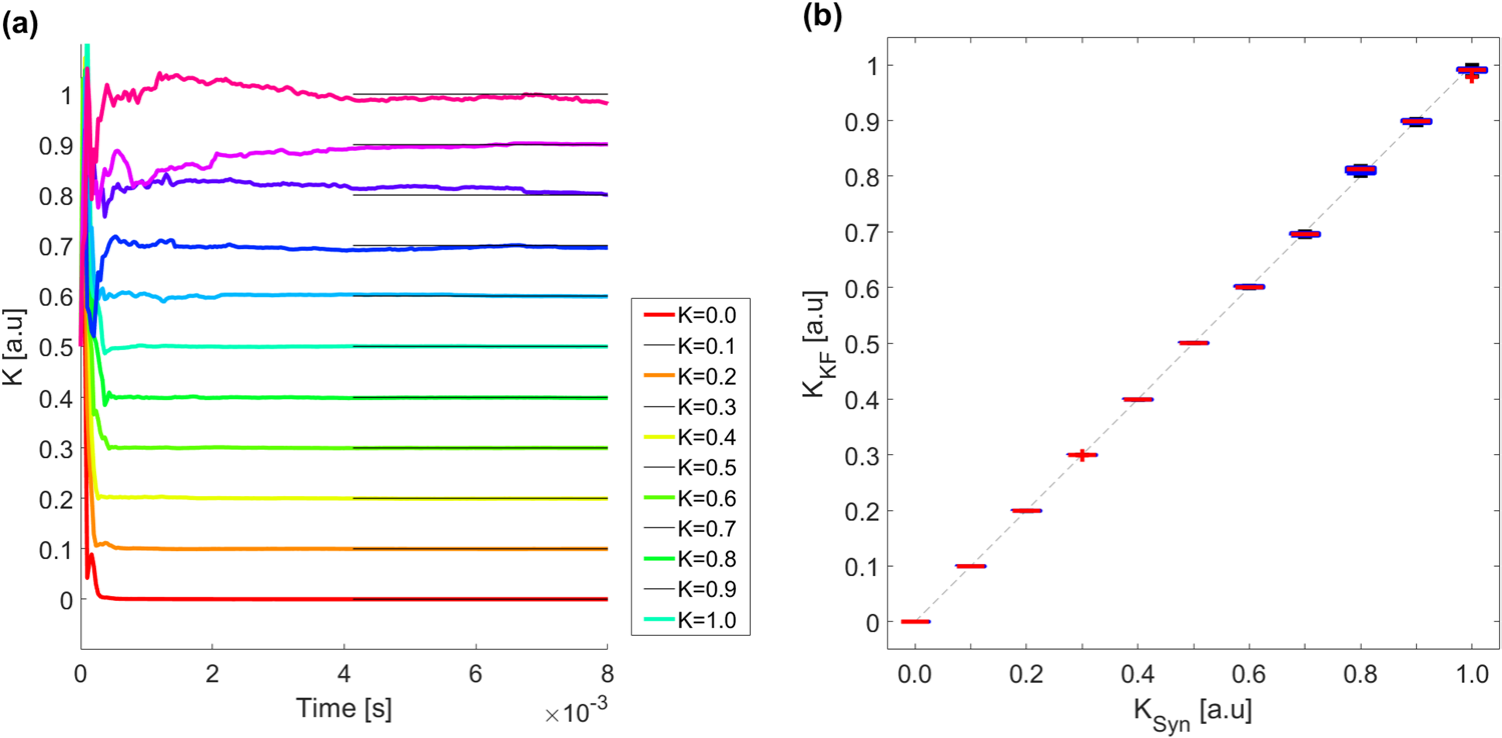
(**a**) UKF estimations obtained from the observations $y_i$ ($i=1,\ldots ,28$) for *K* parameters ranging from 0 to 1. The network of $N=28$ nodes corresponds to the Network 1 as presented in Ref.^41^ and parameters shown in Table 1. In this case, the observation noise is smaller (which makes a faster convergence of the estimations) and the duration of the time traces is shorter than in Fig. 3. The solid black lines indicate the values of the K parameters to be estimated during the filtering. (**b**) Comparison of the estimated and synthetic K using boxplots. For each boxplot, the red line is the median, the upper and lower whiskers of the bars represent the maximum and minimum limits, and the red points refer to outliers. In these simulations, we have used fully stabilized parameters so they show almost perfect estimated values.

Figure 6 presents the results obtained when inferring the value of *K* when we know the network topology, $A_{ij}$. As it can be seen, the UKF quickly recovers the correct value of the coupling parameter. The broadest discrepancies are, again, for high values of *K*. Notice, also, that in these cases, the duration of the time traces are shorter than in Fig. 3. The reason for this is that, here, the level of noise is smaller (see Table 1).

**Figure 7:**
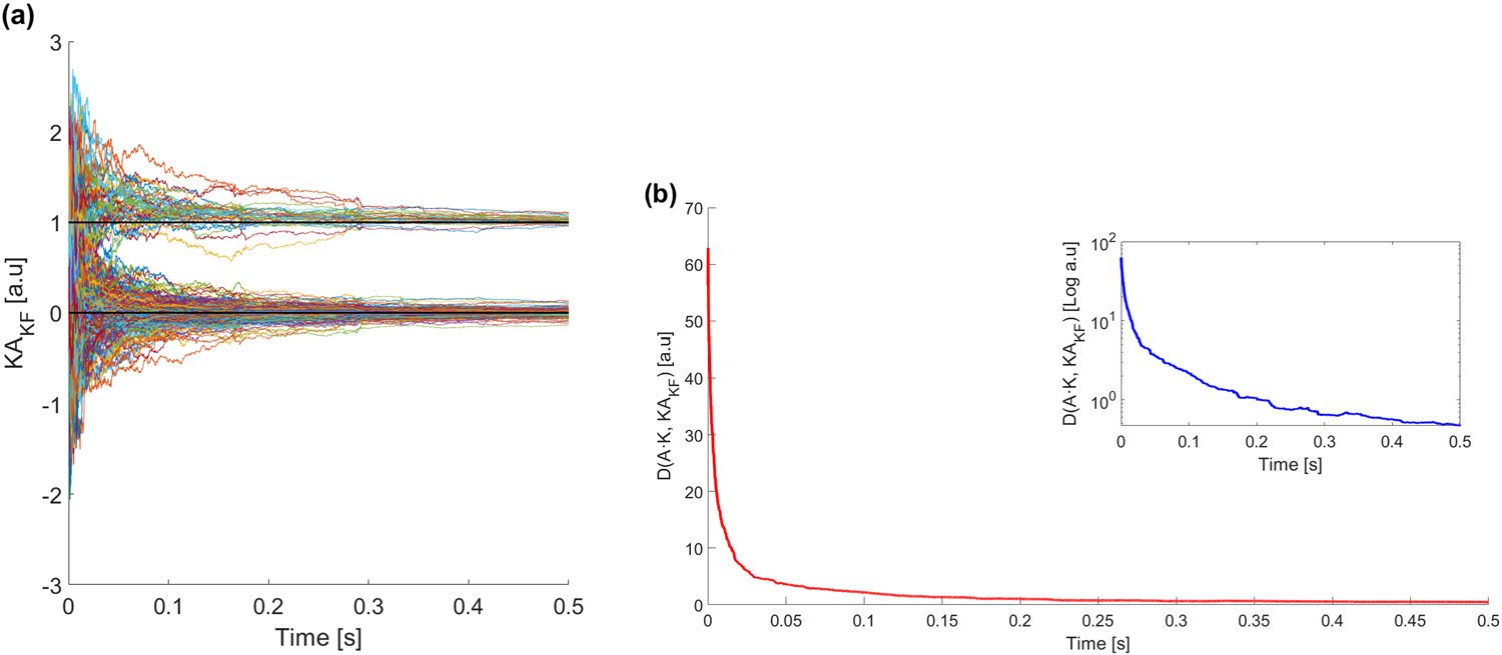
(**a**) Evolution of estimated parameters $K_{ij} \equiv K\cdot A_{ij}$ with $K=1$ for the network of 28 coupled Rössler-like oscillators (see Table 1). The coupling strengths converge to either 0 or 1 representing not connection and connection, respectively, between nodes *i* and *j*. (**b**) The distance between the inferred adjacency matrix and the real one, $D(K,K^{UKF})$, converges to zero. In the inset of panel (**b**) we show the logarithmic evolution of the distance, *D*, showing that all the estimated coupling terms converge to the correct value.

The results obtained when estimating the 378 coupling parameters with $K=1$ are displayed in Fig. 7a. It is clear that the algorithm can distinguish two different populations of $K_{ij}$: one placed around 1 (the links) and another one around 0. Notice that, in this case, longer time traces are needed to obtain full convergence of all the estimated parameters. The reason for this is two-fold: the large number of parameters estimated, and the fact that we use $K=1$, which we have seen is more computationally demanding. In fact, the computational time needed here is about 5000 s contrasting with the few tens of seconds required for the previous results. The main reason for this change is the higher number of parameters to be estimated.

It should be noted that no estimated parameter changes from one group of values to the other (from close to zero and close to one). However, they essentially approach the asymptotic value more or less monotonously. In other words, if we fix a threshold, say, equal to 0.5, promptly we can classify the parameter values into two groups. This allows us to obtain the adjacency matrix, $A_{ij}$, without the burden of long simulations.

To quantify the quality of the network reconstruction we computed the Euclidean distance between the real and reconstructed matrices:

2 $$\begin{aligned} D(K,K^{UKF})=\sqrt{\sum _{i,j}(K_{ij}-K^{UKF}_{ij})^2}. \end{aligned}$$

The results are presented in Fig. 7b, where we see that *D* quickly tends to 0. By forcing the estimated parameters which are, say, above 0.5 to one and forcing the rest of the estimated parameters to zero, we perfectly reconstruct the real adjacency matrix.

## Discussion and conclusions

To summarize, we have shown that the connectivity of a network of coupled oscillators can be inferred by using the Kalman filter technique. In order to do this, a good knowledge of the equations that describe the system is required. However, the level of confidence in this knowledge can be tuned by adjusting the matrix ${\bar{Q}}_{\omega }$. From the experimental side, observed data is required (e.g., $y_i(t)$ for $i=1,\ldots ,N$) and a measurement function that relates the system variable(s) with the observed data is also needed. Again, the level of confidence in this knowledge can be tuned by adjusting the matrix ${\bar{Q}}_{\nu }$.

Specifically, we have shown that the Unscented Kalman Filter (UKF) allows us to infer the connectivity by analyzing the dynamical evolution of only one of the three variables that define the phase space of each individual oscillator. After considering the state estimation of an isolated oscillator, we expanded the study to the minimal motif of two coupled oscillators. Using this approach we show that both the coupling strength and the directionality of connections can be recovered, even if the oscillators’ signals are noisy. For this noisy signals, in order to obtain a better estimate of *K*, it is necessary to run the algorithm for much longer times, which is computationally demanding (especially for large values of *N*). We remark that the network topology can be inferred even when the oscillators are nearly synchronized.

We further tested the method by considering a random network of 28 oscillators. Again, we were able to obtain the coupling strength for the full range of values going from completely uncoupled, $K=0$, to fully coupled, $K=1$, with highly synchronized dynamics. In both situations, $N=2$ and $N=28$, it is observed that the inference of *K* requires more data values for higher synchronization levels. However, if enough data is available, the correct estimation is obtained. As a final proof of the good performance of the method, the correct adjacency matrix was obtained for the network of $N=28$ oscillators for $K=1$.

While the KF technique has important advantages, it also has some limitations. A main advantage is that Kalman filtering requires only partial observation of the state of the system to make predictions. This is an important feature of the method because the capability of inferring variables which are not accessible experimentally is remarkable in cases where experimental observations are limited. A drawback of the methodology is that it requires sufficiently long time traces for the convergence of the estimated values of the parameters. Another important drawback is that it requires a good knowledge of the system, i.e., a good model (with unknown parameters) needs to be used in the prediction step and a good measurement function linking the model to experimental observations is also needed.

As explained in the Introduction, we have used the implementation of Rössler oscillators given in Eqs. (7a–c) because it corresponds to an electronic implementation from which large experimental data sets are available^39,41^. Our objective is to be able to recover the experimental couplings. However, to do so, more information of the system is required. We have tried to infer connectivities from the experimental data sets presented in^39,41^; however, the application of the UKF algorithm to simulations using Eqs. (7a–c) with the parameters indicated in^39,41^ failed to provide a good agreement between the simulated time series and the experimentally measured ones. This may be due to typos in the parameter values and/or it might be due to the filtering of the signals performed by the data acquisition system. Improved knowledge of the experimental setup and data-acquisition methods is required for obtaining, using our method, a good reconstruction of the network.

It will be interesting, for future work, to apply the KF technique to uncover the connectivity of a network that evolves in time, where, for example, the strength of the connections varies. It will also be appealing to study the suitability of the KF technique when the observed data is heavily contaminated by noise. The results obtained here are promising and justify trying to use this approach in different scenarios.

## Methods

### Kalman filter

The KF, in its different implementations, is a standard tool used to estimate the unknown state of a system using a sequence of discrete-time observations and a model of the system evolution. Additionally, the filter can be used to infer a parameter value^33^ by considering it as an additional system variable with the trivial dynamic evolution (being constant).

Originally, the filter was created for linear dynamical systems assuming additive Gaussian noise processes adding uncertainty to the state evolution and the observations. In this way, the algorithm makes recursive optimal estimates of the state of the system and its parameters^30^. The filter was soon extended to be able to deal with nonlinear systems^42^. Here, we use the Unscented Kalman Filter^43,44^ implemented using the package *unscentedKalmanFilter* from *Matlab*.

The assumption underlying the UKF is that the system evolves as a noisy first-order Markov process

3 $$\begin{aligned} \bar{{\mathbf {u}}}_{k+1}=\bar{{\mathbf {a}}}(\bar{ {\mathbf {u}}}_k) + \bar{\mathbf \omega }_k, \end{aligned}$$

where ${\bar{\mathbf{u }}}_k$ corresponds to the state at the discrete-time *k*, ${\bar{\mathbf{a }}}$ is the vectorial function that maps the state at time *k* to the state at the next time, $k+1$, and ${\bar{\omega }}_k$ is the dynamic (or process) noise vector. The system is measured at each time *k* applying a measurement function ${\bar{\mathbf{b }}}$ to the state ${\bar{\mathbf{u }}}_k$, perturbed by the measurement noise ${\bar{\nu }}_k$:

4 $$\begin{aligned} \bar{{\mathbf {w}}}_{k}=\bar{{\mathbf {b}}}(\bar{{\mathbf {u}}}_k)+{\bar{\nu }}_k. \end{aligned}$$

Both the dynamic noise ${\bar{\omega }}_k$ and the measurement noise ${\bar{\nu }}_k$ are additive zero-mean Gaussian processes with covariance $\bar{Q}_{\omega }$ and $\bar{Q}_{\nu }$, respectively.

The algorithm works as follows. The estimate of the hidden state of the system ${\mathbf {u}}_k$ at a given time is obtained in two steps. First, a new prediction is done, using $\bar{{\mathbf {a}}}$ and the previous estimate of the state. The information carried by the system observations, $\bar{{\mathbf {w}}}_{k+1}$, is then integrated with the prediction. The result is a prediction–correction algorithm that estimates the state $\bar{{\mathbf {u}}}_{k+1}$. The algorithm also returns an estimate of the state covariance matrix, which provides the uncertainty on the estimation of $\bar{{\mathbf {u}}}_{k+1}$.

The uncertainty of the model prediction and of the experimental information is represented by the covariances of the stochastic terms ${\bar{\omega }}_i$ and ${\bar{\nu }}_i$. The filter is run using initial guesses of these two terms, so, by choosing them appropriately, the uncertainty of the model and of the experimental measurements is adjusted.

In the case in which *q* models parameters, $(p_1,\dots , p_q)$ have to be estimated, the hidden state is extended to incorporate them as additional variables:

5 $$\begin{aligned} \bar{{\mathbf {u}}}_e=\left( u_1, \dots , u_n, p_1, \dots , p_q\right) \end{aligned}$$

The trivial evolution of the parameters is governed by $\dot{p}_i=0$, without process noise, resulting in an extended covariance matrix^33^:

6 $$\begin{aligned} \bar{Q}_{\omega ,e}= \begin{pmatrix} Q^{\omega }_{11} &{} \ldots &{} Q^{\omega }_{1n} &{} 0_{1,n+1} &{} \ldots &{} 0_{1,n+q} \\ Q^{\omega }_{2,1} &{} \ldots &{} Q^{\omega }_{2,n} &{} 0_{2,n+1} &{} \ldots &{} 0_{2,n+q} \\ Q^{\omega }_{3,1} &{} \ldots &{} Q^{\omega }_{3,n} &{} 0_{3,n+1} &{} \ldots &{} 0_{3,n+q} \\ \vdots &{} \vdots &{} \vdots &{} \vdots &{} \ddots &{} \vdots \\ Q^{\omega }_{n,1} &{} \ldots &{} Q^{\omega }_{n,n} &{} 0_{n,n+1} &{} \ldots &{} 0_{n,n+q} \\ 0_{n+1,1} &{} \ldots &{} 0_{n+1,n} &{} 0_{n+1,n+1} &{} \ldots &{} 0_{n+1,n+q} \\ \vdots &{} \vdots &{} \vdots &{} \vdots &{} \ddots &{} \vdots \\ 0_{n+q,1} &{} \ldots &{} 0_{n+q,n} &{} 0_{n+q,n+1} &{} \ldots &{} 0_{n+q,n+q} \\ \end{pmatrix}. \end{aligned}$$

This matrix is the extended form presented in Eq. (1), but now for *q* unknown parameters. The terms $Q^\omega _{ij}$ correspond to the elements of the covariance matrix obtained from the noise term $\omega _k$ in Eq. (3) for the *n* dynamical variables of the system. We estimate these terms using a realization of the dynamical noise shown in Eq. (3). The rest of the terms of the matrix are zeros, corresponding to the covariance of the *q* constant parameters to be adjusted by the algorithm.

### Models and data sets

In this work, we simulated networks composed of *N* Rössler-like oscillators described by Eqs. (1)–(4) in Sevilla-Escoboza et al^39,41^. The equations describing the system are the following:

7a $$\begin{aligned}&\dot{x}_i = -\alpha _1 \left( x_i + \beta y_i + \Gamma z_i \right) , \end{aligned}$$

7b $$\begin{aligned}&\dot{y}_i = -\alpha _2 \left( - \gamma x_i + \left( 1 - \delta \right) y_i -K\phi \sum _{j=1}^N A_{ij}\left( y_j - y_i\right) \right) , \end{aligned}$$

7c $$\begin{aligned}&\dot{z}_i = -\alpha _3 \left( -\eta G_{x_i} + z_i\right) , \end{aligned}$$

where

8 $$\begin{aligned} G_{x_i} = {\left\{ \begin{array}{ll} 0 &{} \mathrm {if}~~ x_i\le V_{{th}} \\ \mu (x_i - V_{th}) &{} \mathrm {if}~~ x_i > V_{{th}} \end{array}\right. }. \end{aligned}$$

We used the same values for all the parameters as in^39^, except for $\alpha _3=10{,}000$^45^. Details about the value of the parameters can be found in Table 1. Experimental time series of the variable $y_i$ ($i=1,\ldots ,N$) for each oscillator of the network are freely available for a wide range of coupling strengths and topologies^39,41^. Here, as a proof-of-concept, we applied an UKF to simulated time series of the variables $y_i$ to infer the state of the oscillators and their connectivity under the assumption that we have a precise knowledge of the governing equations of the individual elements of the system. In the future, using these results as a starting point, we will try to reconstruct the networks from the available experimental data. However, this requires incorporating the manipulations performed during the data acquisition into the measurement function of the UKF (Eq. 4), since the available experimental data is not faithfully represented only by the raw solution of the Eqs. (1)–(4) in Ref.^39^.

To use the UKF, we transform this set of equations to the form defined in Eq. (3) which includes the presence of noise in the system ($\omega ^i_x(t_k),\omega ^i_y(t_k),\omega ^i_z(t_k)$). From this respect, the function $\bar{{\mathbf {a}}}$ is a Runge-Kutta step of fourth-order and a time-step *dt* defined in Table 1, while ${\bar{\omega }}$ are Gaussian stochastic processes having zero mean and standard deviation $\sigma _\omega$ (see Table 1). From realizations of these noise terms we estimate the covariance ${\bar{Q}}_{\omega }$ used in the filter algorithm.

**Table 1:** Parameters used in the simulations of Eqs. (7a–c) for the isolated node, a couple of nodes and the network of 28 nodes configurations. These parameters correspond to those used in the experimental set-up presented in Refs.^39,41^, respectively.

Parameter	N=1, 2	N=28
$\alpha _1$	500 s$^{-1}$	500 s$^{-1}$
$\alpha _2$	200 s$^{-1}$	200 s$^{-1}$
$\alpha _3$	10,000 s$^{-1}$	10,000 s$^{-1}$
$\beta$	10	10
$\Gamma$	20	20
$\gamma$	50	50
$\delta$	8.3333	8.6207
$\phi$	10.02	10
$\eta$	1	1
$\mu$	15	15
$V_{th}$	3.0088 *V*	2.1265 *V*
*K*	[0, 1]	[0, 1]
$\sigma _{\omega }$	0.02	0.001
$\sigma _{\nu }$	0.5	0.001
$f_s$	37 KS/s	30 KS/s
$dt=1/f_s$	2.7027e−05 s	3.3333e−05 s
*n* number of time steps	100,000	250, 15,000

Finally, to mimic a measurement process, we perturbed the $y_i$ variable with a Gaussian stochastic term as defined in Eq. (4) having zero mean and standard deviation $\sigma _\nu$ (see Table 1). We decided to measure only variable *y* for two reasons: (i) in the experiments in^39^, *y* was the recorded variable for each oscillator, (ii) the coupling between the oscillators is reached by coupling their $y_i$. This choice corresponds to $\bar{{\mathbf {b}}}(x_1, y_1, z_1, \ldots , x_N, y_N, z_N) = (y_1, y_2, \ldots , y_N)$ in Eq. (4).

In Eqs. (7a–c), the oscillators are mutually coupled with identical coupling strength, *K*. The coupling topology, for the different networks considered, is described by the adjacency matrix *A* defined as follows: $A_{ij}=A_{ji}=1$ if oscillators *i* and *j* are connected, and $A_{ij}=A_{ji}=0$ if they are not. There are no feedback loops, so we take $A_{ii}=0$. In the case of $N=2$ oscillators, to recover the coupling strength, we used the UKF to infer the value of one parameter only, *K*, and to reconstruct the directionality of the coupling, we used the UKF to infer the values of two parameters: $K_{12}=A_{12} \cdot K$ and $K_{21}=A_{21} \cdot K$. For the analysis of $N=28$ oscillators (in this case parameters are the same as in^41^), we considered two different scenarios. First, we assumed that we know the adjacency matrix *A*, and we tried to infer the common coupling strength *K*, which is the same for all connections. In the second situation, we assumed bidirectionality and we tried to infer the $N(N-1)/2=378$ unknown elements of $K \cdot A_{ij}$.
